# 

**Published:** 1902-02-15

**Authors:** 


					﻿the
DENTAL REGISTER
EDITED BY
NELVILLE S. HOFF, D. D. S.,
ANN ARBOR, MICH.
Vol. LVI. FEBRUARY 15, 1902.	No. 2.
PRICE, ONE DOLLAR A YEAR IN ADVANCE.
PUBLISHED MONTHLY BY
SAMIJEI. A, CROCKER & CO.,
THE OHIO DENTAL AND SURGICAL DEPOT.
3.” to :?S> W. “tli JSt., Cincinnati, O.
Entered at the Postoffice at Cincinnati, 0., as second-class mail matter.
				

